# A social cognitive theory and theory of planned behavior–based serial multiple mediation model of social media–related factors and eating behaviors among sports science students

**DOI:** 10.3389/fnut.2026.1847236

**Published:** 2026-06-09

**Authors:** Erhayat Özgür Bayazıtlı, Ayşe Sena Çakır, Mehmet Behzat Turan, Uğur Caba, Barış Karaoğlu, Osman Pepe, Aydın Pekel, İncinur Hanik Sevim, İbrahim Dalbudak

**Affiliations:** 1 Department of Sports Management, Faculty of Sports Sciences, İstanbul Gelişim University, İstanbul, Türkiye; 2 Department of Health Management, Develi Faculty of Social and Human Sciences, Kayseri University, Kayseri, Türkiye; 3 Department of Recreation, Faculty of Sports Sciences, Erciyes University, Kayseri, Türkiye; 4 Department of Exercise and Sports Sciences, Faculty of Sports Sciences, İstanbul Gelişim University, İstanbul, Türkiye; 5 Department of Recreation, Faculty of Sports Sciences, Bingöl University, Bingöl, Türkiye; 6 Department of Sports Management, Faculty of Sports Sciences, Süleyman Demirel University, Isparta, Türkiye; 7 Department of Sports Management, Faculty of Sports Sciences, Marmara University, İstanbul, Türkiye; 8 Department of Sports Management, Faculty of Sports Sciences, Uşak University, Uşak, Türkiye

**Keywords:** acceptance of food cravings, digital nutrition literacy, eating behavior, emotional eating, functional foods, serial multiple mediation, social media

## Abstract

**Background/objectives:**

Social media has become a pervasive digital environment closely associated with individuals’ dietary preferences, eating attitudes, and consumption behaviors. Although prior research has documented associations between social media use and eating behaviors, the underlying cognitive, attitudinal, and psychological mechanisms remain insufficiently clarified, particularly among university students. Accordingly, this study examines the serial mediating roles of digital healthy nutrition literacy, attitudes toward functional foods, food craving acceptance, and emotional eating in the relationship between social media use and eating behaviors.

**Methods:**

This cross-sectional study was conducted with 1,576 sports sciences students from 38 public universities in Türkiye. Sports science students were selected because of their high engagement with health-related content, intensive social media exposure, and potential future role in promoting healthy lifestyle behaviors. Data were collected using validated instruments. The proposed serial multiple mediation model was tested using PROCESS Macro (Model 6) with 5,000 bootstrap resamples.

**Results:**

Social media–related factors were significantly associated with e-healthy diet literacy (*β* = 0.31, *p* < 0.001), attitudes toward functional foods (*β* = 0.31, *p* < 0.001), food craving acceptance (*β* = −0.12, *p* < 0.001), and emotional eating (*β* = 0.12, *p* < 0.001). In turn, e-healthy diet literacy (*β* = 0.27, *p* < 0.001), attitudes toward functional foods (*β* = 0.21, *p* < 0.001), food craving acceptance (*β* = 0.29, *p* < 0.001), and emotional eating (*β* = 1.32, *p* < 0.001) were all significantly associated with eating attitudes. The model explained 50% of the variance in eating attitudes (R^2^ = 0.50). The full serial pathway was statistically significant, although the indirect effect size was small (B = 0.01, 95% CI [0.01, 0.01]). The total indirect effect was statistically significant (B = 0.51, SE = 0.04, 95% CI [0.44, 0.58]), while the full serial mediation pathway also reached significance (B = 0.01, 95% CI [0.01, 0.01]).

**Conclusion:**

The findings indicate that the relationship between social media use and eating behaviors involves a sequence of cognitive, attitudinal, and emotional processes rather than a single direct pathway. Digital healthy nutrition literacy and acceptance-based regulation processes appear to play key roles within this framework. Given their intensive social media use and potential role-model status, sports sciences students represent an important target group for such approaches.

## 1 Introduction

Social media is an umbrella term encompassing diverse online platforms, including blogs, forums, microblogs, video-sharing services, and virtual environments (1). With its rapid global expansion, it represents a pervasive digital environment closely associated with individuals’ eating preferences, meal patterns, and consumption contexts (2). This association is particularly evident through visually driven content and algorithm-based systems that personalize exposure to food-related information (3). Engagement with food-related content including cooking videos, mukbang/cookbang broadcasts, and recipe streams has been associated with eating frequency and broader dietary patterns. However, the magnitude and direction of these relationships may vary depending on content characteristics (3). Notably, highly appealing food content has also been associated with increased appetite and less healthy eating behaviors (4).

These patterns can be understood within Social Cognitive Theory, which emphasizes observational learning, modeling, and social comparison (5). Social media provides a salient context in which users are repeatedly exposed to modeled eating behaviors, shaping perceptions of social norms and expectations (6). Continuous exposure to curated food content may recalibrate perceptions of what is normative or desirable eating, which, in turn, is associated with behavioral tendencies.

Within the framework of Social Cognitive Theory, social media influencers, peers, and digital content creators can function as symbolic role models whose dietary practices and food-related behaviors are observed, imitated, and potentially internalized by users through repeated exposure (7, 8).

However, the relationship between social media use and eating behaviors extends beyond exposure alone. Individuals’ ability to access, evaluate, and apply nutrition-related information in digital environments is key. Digital healthy eating literacy is closely associated with how such content is interpreted and translated into behavior. Supporting this view, evidence suggests that health literacy and digital healthy eating literacy are important factors associated with the link between social media use and healthy eating patterns (9). Individuals with higher literacy appear better equipped to evaluate online content and make informed dietary decisions critically.

Accordingly, digital healthy eating literacy can be conceptualized as a central cognitive mechanism associated with this relationship. In parallel, functional foods defined as foods providing additional health benefits beyond basic nutrition play an important role in health promotion (10). Social media platforms are increasingly used to promote these products, shaping consumer perceptions and preferences (11), and social media engagement has been associated with functional food consumption (12).

The literature also indicates that social media use is associated with disordered eating behaviors and related cognitive-emotional processes (13, 14). Increased engagement has been linked to emotional eating and heightened craving responses, particularly in visually rich environments (14, 15). Similarly, food-focused social media exposure has been associated with indicators of disordered eating and higher emotional eating levels in certain groups (16).

Within this context, acceptance of food cravings refers to the ability to experience cravings without suppression (17). Acceptance-based approaches are associated with improved self-regulation, whereas suppression strategies may intensify maladaptive responses. In stimulus-rich environments such as social media, acceptance processes are associated with reduced impulsive eating and more adaptive regulation. The growing body of research further highlights increasing scholarly attention to the relationship between social media and dietary behaviors (18–20).

Recent systematic reviews and bibliometric analyses further demonstrate a substantial increase in research examining the associations between social media exposure, dietary patterns, emotional eating, and nutrition-related behaviors, particularly among adolescents and young adults (21, 22).

Despite this expansion, existing studies often focus on isolated variables. Integrative approaches that simultaneously examine cognitive (digital healthy eating literacy), attitudinal (functional food attitudes), and psychological (food craving acceptance) mechanisms remain limited. This suggests that the pathways through which social media use is associated with eating behaviors have not been sufficiently addressed within a unified framework.

Importantly, although the literature distinguishes among different types of food-related social media content, the present study adopts a general approach, focusing on the overall perceived association between social media and eating behavior. The measurement instrument captures subjective exposure and perceived impact, but does not differentiate between content types. Therefore, the findings should be interpreted within the context of generalized social media use rather than specific mechanisms.

Furthermore, although digital inequalities and health disparities are highly relevant, the present study does not include direct measures of socioeconomic status or structural inequities. Accordingly, it focuses on individual-level cognitive, attitudinal, and psychological mechanisms associated with eating behavior (23, 24).

Accordingly, the present study examines the relationship between social media use and eating behaviors through a serial mediation model incorporating digital healthy eating literacy, attitudes toward functional foods, and acceptance of food cravings. By integrating cognitive, attitudinal, and psychological processes, the study provides a comprehensive perspective on how social media is associated with eating behavior.

Unlike prior research focusing on isolated relationships, this study proposes a sequential mechanism including cognitive, attitudinal, and psychological regulation processes. To our knowledge, it is the first to test a full serial multiple-mediation model linking social media use to eating behavior through these interrelated mechanisms in a large, multi-university sample. By modeling these processes as a dynamic system, the study contributes to theory development at the intersection of digital health, nutrition, and behavioral science.

To maintain conceptual clarity, the study prioritizes literature directly related to student populations and health-oriented groups, thereby refining the research gap and avoiding redundancy in broader social media research.

### 1.1 Social media use

Social media constitutes a dynamic digital environment that facilitates interaction within human networks. Encompassing activities such as messaging, information seeking, and content browsing, it refers to internet-based platforms that enable individuals to communicate, interact, and construct their identities in online contexts (25, 26). Young adults aged 18–29 represent the most active group of users, underscoring the central role of social media in daily experiences and behavioral patterns within this population (27, 28).

Health- and nutrition-related content is among the most frequently shared domains on these platforms and is closely associated with individuals’ dietary choices and consumption patterns (6). However, a substantial proportion of this information lacks a scientific basis and may include misleading claims (29). Exposure to such content has been associated with maladaptive dietary patterns, highlighting the importance of evaluating the credibility of online information (30, 31). In this context, the ability to critically appraise digital health and nutrition content emerges as a key competency associated with informed dietary decision-making.

At the same time, the relationship between social media use and health-related outcomes is not unidirectional. Under certain conditions, social media can function as a supportive learning environment. For example, Sercu (32) reported that adolescents who more frequently followed influencers sharing healthy lifestyle content exhibited higher levels of health and nutrition literacy. Improvements were particularly evident in functional and critical literacy, although no significant differences were observed in objective health knowledge. These findings suggest that exposure to credible content is associated with enhanced literacy, even if it does not directly translate into gains in factual knowledge.

Similarly, research conducted among women in rural areas indicated that both the duration and purpose of social media use are associated with health literacy (HL) and digital healthy eating literacy (DDL). Individuals who used social media more frequently, particularly for informational purposes, demonstrated higher HL and DDL levels. These literacy levels were positively associated with adherence to the Mediterranean diet and negatively associated with body mass index (BMI) (9). Overall, these findings suggest that purposeful engagement with accurate content is associated with the development of health-related competencies and healthier behavioral patterns.

In the present study, social media use is conceptualized as a general behavioral construct encompassing exposure to food-related content across multiple widely used platforms (e.g., Instagram, TikTok, and YouTube). However, the study does not differentiate among specific platforms; instead, it focuses on the overall perceived association between social media environments and eating behavior rather than platform-specific effects.

This approach was adopted to capture the broader perceived influence of social media environments on eating-related tendencies rather than isolating platform-specific algorithmic or content-related effects. Nevertheless, platforms such as Instagram, TikTok, and YouTube are considered particularly relevant because of their visually oriented food-related content structures (3, 20).

### 1.2 Digital healthy eating literacy

Nutritional literacy refers to individuals’ ability to obtain, process, and understand essential nutrition information needed to make appropriate dietary decisions (33). Within this framework, the capacity to access, interpret, and apply nutrition knowledge is associated with food choices and overall dietary behaviors (34, 35).

With the growing reliance on digital platforms for health and nutrition information, digital nutritional literacy has become increasingly important (36). However, the abundance of inconsistent and sometimes misleading online content presents challenges, making the ability to identify accurate and reliable information a critical competency (37). Digital health literacy is therefore conceptualized as a multidimensional construct encompassing not only access but also understanding, evaluation, and effective use of information (31, 38). Expanding this view, the Transactional eHealth Literacy Model highlights individuals’ capacity to locate, evaluate, share, and apply online health information, particularly emphasizing its translation into real-life behaviors (39).

Health behavior theories further suggest that factors such as self-efficacy, perceived usefulness, and perceived control are associated with how individuals interpret and act on health information (40). Accordingly, individuals with higher digital nutritional literacy are more likely to trust credible sources and feel greater control over their dietary decisions, which are associated with healthier, more sustainable eating behaviors. Empirical evidence from Turkey supports this, showing that higher e-healthy diet literacy (e-HDL) is associated with healthier food choices and greater adherence to the Mediterranean diet (41), as well as improvements in sustainable eating behaviors among university students (42).

Functional foods, often evaluated based on perceived benefits, trust, and knowledge, are also associated with consumers’ cognitive evaluations (43). Supporting this, nutritional literacy is positively associated with attitudes toward functional foods (44). In this context, digital nutritional literacy may similarly shape these attitudes, as individuals who can effectively interpret online nutrition information are more likely to develop informed and potentially more favorable perceptions of such products.

### 1.3 Attitudes toward functional foods

Functional foods are defined as foods that provide health benefits beyond basic nutrition by positively affecting physiological functions and reducing disease risk (45). They are typically enriched with bioactive compounds or live microorganisms at effective levels to promote health. These components include nutrients, dietary fiber, phytochemicals, and probiotics (46). Common examples are omega-3–enriched products, probiotic foods such as yogurt and kefir, prebiotic formulations, and foods fortified with plant sterols, as well as naturally occurring compounds like polyphenols with antioxidant properties (46).

According to the Theory of Planned Behavior, attitude reflects an individual’s positive or negative evaluation of a behavior and is a key determinant of intention (47). Attitudes toward functional foods are influenced by multiple factors, including product characteristics, psychological traits, trust in information sources, and perceptions of novelty (43). Evidence indicates that favorable attitudes are associated with stronger consumption intentions, particularly when supported by health awareness and trust in food-related information (48).

However, intentions do not always translate into behavior due to barriers such as cost, accessibility, and misinformation, leading to the “intention behavior gap” (48). Thus, positive attitudes toward functional foods may not consistently result in actual consumption (43, 48).

In this process, self-regulation plays a critical role. Food cravings are associated with unhealthy eating and deviations from intended behaviors (49, 50). Higher levels of awareness and acceptance are associated with reduced cravings, while mindfulness-based approaches have been shown to attenuate these tendencies (49, 50). Accordingly, although positive attitudes toward functional foods may foster healthy intentions, their translation into behavior depends on individuals’ ability to accept and regulate food-related urges.

### 1.4 Acceptance of food cravings

A food craving is defined as an intense and specific desire to consume a particular food, distinguishing it from general hunger, which can be satisfied with various options (51, 52). This specificity reflects the psychological and hedonic nature of cravings beyond basic physiological needs.

Food cravings are multidimensional, involving cognitive, emotional, behavioral, and physiological components (53). To regulate these urges, individuals often use control-based strategies such as suppression or distraction (17). However, such approaches may have limited long-term effectiveness.

Acceptance-based approaches, in contrast, emphasize experiencing cravings without resistance (54). Attempts to suppress internal states may intensify them, as cravings involve recurring thoughts, perceived loss of control, and emotional engagement, increasing the likelihood of food intake under reduced self-regulation (55). Acceptance-based strategies aim to enhance psychological flexibility and reduce experiential avoidance, allowing individuals to acknowledge cravings without acting on them (56–58).

Empirical evidence shows that such approaches are associated with reduced energy intake and maladaptive eating responses (58, 59). While suppression is associated with overeating and emotional eating, mindfulness- and acceptance-based interventions are associated with reductions in both emotional and external eating behaviors, supporting their role as adaptive self-regulation strategies (60, 61).

### 1.5 Emotional eating

Emotional eating is defined as the tendency to consume energy-dense, nutrient-poor foods in response to negative emotions such as stress, depression, and loneliness, rather than to physiological hunger (62, 63). This pattern reflects an emotion regulation strategy in which food is used as a coping mechanism to alleviate negative affect.

The relationship between emotion and eating is multidimensional and varies across individuals (64). Although negative emotions may suppress intake in some cases, they are associated with increased hedonic consumption among individuals prone to emotional eating (65, 66). This suggests that emotional eating is shaped by learned associations and prior experiences linking emotions to food-related behaviors (67).

Large-scale evidence indicates that emotional eating is associated with emotional states such as stress, depressive mood, loneliness, and boredom, as well as with motives such as feeling good and relaxing (68). These findings highlight that eating behavior is closely tied to emotional regulation rather than solely to biological need.

Over time, repeated use of food for emotional relief reinforces conditioned patterns in which emotional cues trigger eating even in the absence of hunger (63, 64). From a neuropsychological perspective, negative emotional states are associated with increased reward sensitivity to high-calorie foods and heightened activation in reward-related brain regions (69). At the same time, these states are associated with reduced cognitive control, biasing decision-making toward immediate gratification and increasing the likelihood of emotionally driven eating behaviors (69).

### 1.6 Eating behavior

Eating behavior is a complex construct reflecting the interaction of psychological, physiological, genetic, and social factors that shape food preferences, intake, and dietary choices (70). It is therefore not only driven by biological needs but also associated with cognitive, emotional, and environmental determinants.

The literature conceptualizes eating behavior as multidimensional, including cognitive, emotional, and behavioral components. The Three-Factor Eating Questionnaire (TFEQ) identifies cognitive restraint, disinhibition, and hunger (71), while its revised version (TFEQ-R18) includes cognitive restraint, uncontrolled eating, and emotional eating (72). This framework indicates that eating behavior is associated with both deliberate regulation and impulsive, emotion-driven processes.

In stimulus-rich environments, eating behavior is increasingly associated with hedonic processes. Visually appealing food cues may trigger “visual hunger,” reflecting a desire to eat in the absence of physiological need (14). Supporting this, research shows that exposure to food-related content on social media is associated with eating behaviors and patterns (3, 73). These findings suggest that digital environments are associated with eating behavior through cue-driven and hedonic mechanisms.

Within this context, eating behavior emerges from the interaction of cognitive, attitudinal, and emotional factors. Digital healthy eating literacy is associated with more informed food choices by enabling critical evaluation of online information. Similarly, positive attitudes toward functional foods are associated with healthier intentions. However, translating these intentions into behavior depends on regulatory and affective processes, such as food cravings and emotional eating.

Accordingly, eating behavior can be conceptualized as a multidimensional outcome situated at the intersection of cognition, attitudes, and emotional regulation, underscoring the need for integrative approaches to understand its underlying mechanisms better.

### 1.7 The present study

Although previous studies have examined several isolated associations among social media use, nutrition literacy, emotional eating, and food-related attitudes, the existing literature remains fragmented. The present review therefore selectively focuses on evidence directly relevant to the proposed serial mediation model and the unique characteristics of sports science students in order to establish a clearer theoretical and empirical rationale for the current study.

Although many studies have examined the relationship between social media use and eating behaviors, integrative models explaining the underlying cognitive and psychological mechanisms remain limited. Most research focuses on direct associations with outcomes such as eating patterns or unhealthy dietary tendencies (3, 73), while studies adopting a holistic perspective that links exposure to nutritional content with cognitive evaluation, attitudinal formation, and emotion regulation are scarce. This gap highlights the need for more comprehensive frameworks.

Digital nutritional literacy, defined as the ability to access, evaluate, and use nutrition information in digital environments, is a key cognitive mechanism associated with how social media content is interpreted and translated into behavior (36, 37). However, knowledge alone is insufficient; attitudinal factors, particularly attitudes toward functional foods, are also associated with dietary choices (43, 48). The extent to which these attitudes translate into behavior depends on self-regulatory processes, such as managing food cravings.

Acceptance of food cravings represents an important regulation mechanism, referring to the ability to experience urges without suppression (17). Such strategies are associated with reduced emotional eating (58, 60). Emotional eating itself, defined as eating in response to negative emotions, is consistently associated with unhealthy dietary patterns (62, 63). Together, these findings suggest that eating behavior is associated with the interaction of cognitive, attitudinal, and emotional processes.

Despite this, models integrating digital healthy eating literacy, functional food attitudes, food craving acceptance, and emotional eating in a sequential framework are scarce. Accordingly, this study addresses this gap by examining their chain mediating roles within a unified model, aiming to explain how social media use is associated with eating behaviors through interconnected mechanisms.

The sample consists of university students in physical education and sport sciences, a group highly engaged with social media (27) and at a critical stage for establishing health behaviors (73). Their nutrition awareness and potential role as future health role models make them particularly relevant.

In addition, sports science students are frequently exposed to body image ideals, performance-oriented nutrition messages, and fitness-related digital content through social media platforms. These characteristics may increase their susceptibility to food-related online influences while simultaneously positioning them as future professionals who may shape public perceptions regarding health and nutrition behaviors (74, 75).

This study investigates how social media use is associated with eating behaviors through the mediating roles of digital healthy eating literacy, attitudes toward functional foods, acceptance of food cravings, and emotional eating. The central research question is:

Do digital healthy eating literacy, attitudes toward functional foods, acceptance of food cravings, and emotional eating sequentially mediate the relationship between social media use and eating behaviors?

The proposed model assumes that social media use is associated with eating behaviors both directly and indirectly through cognitive, attitudinal, and psychological mechanisms. By adopting this multidimensional perspective, the study aims to contribute to the literature by providing a comprehensive explanation of how digital environments are associated with dietary outcomes.

### 1.8 Theoretical framework

The rapid expansion of digital technologies has transformed how individuals access and engage with nutrition information. Social media platforms have become major sources of dietary content, particularly among young adults, offering both opportunities for health promotion and risks related to misleading or non-evidence-based information. Exposure to such content has been associated with eating behaviors, food preferences, and diet-related attitudes (76, 77).

To explain these associations, the present study draws primarily on Social Cognitive Theory (SCT) and the Theory of Planned Behavior (TPB).

TPB was incorporated because the proposed model includes attitudinal mechanisms, particularly attitudes toward functional foods, which are conceptualized as central determinants of behavioral tendencies within the TPB framework. Thus, SCT was used to explain observational learning processes associated with social media exposure, whereas TPB was employed to explain how cognitive evaluations and attitudes may translate into eating-related tendencies (47).

It is conceptually informed by the Social Ecological Model (SEM). SCT posits that behaviors are acquired through observational learning within social environments (5), whereas TPB emphasizes attitudes, subjective norms, and perceived behavioral control as key determinants of behavior (47). However, digital food environments are inherently multi-layered. In this regard, SEM highlights the interaction between individual, interpersonal, organizational, and environmental factors (78), providing a broader contextual lens for interpreting digital influences.

The inclusion of the Social Ecological Model (SEM) is particularly relevant because food-related digital environments are shaped not only by individual cognitive processes but also by peer interaction, institutional culture, commercial marketing systems, and broader sociocultural norms. Accordingly, SEM complements SCT and TPB by situating individual dietary decision-making within broader environmental and structural contexts (78, 79).

Within this integrated framework, social media can be conceptualized as a dynamic, multi-level environment in which dietary norms, expectations, and modeled behaviors are continuously observed and interpreted. Social media platforms operate across multiple levels of influence: they shape individual knowledge and attitudes, facilitate peer interaction, and reflect broader cultural and commercial food environments. While the empirical model of the present study focuses on individual-level mechanisms, integrating SCT with SEM allows for a more comprehensive interpretation of how digital exposures are associated with eating behaviors.

From a cognitive perspective, digital nutrition literacy (e-healthy diet literacy) is a key mechanism in how individuals evaluate online information and translate it into behavioral tendencies (76). Individuals with higher literacy levels appear better equipped to critically appraise digital content and engage in more informed dietary decision-making.

From an attitudinal perspective, attitudes toward functional foods are closely associated with dietary preferences, particularly when such foods are perceived as beneficial (80). Within the TPB framework, these attitudes constitute a central component linking cognitive evaluations to behavioral tendencies.

From a psychological perspective, acceptance of food cravings reflects a dimension of self-regulation, particularly relevant in digital environments where exposure to appealing food content is associated with increased cravings (58). In contrast to suppression-based strategies, acceptance-based processes are associated with more adaptive regulation of eating behavior.

Emotional eating represents an additional affective mechanism, characterized by eating in response to negative emotional states and associated with maladaptive dietary patterns. Social media exposure, particularly to food- and body-related content, has been associated with emotional distress and emotional eating (41). These findings highlight the combined role of self-regulation and emotional processes.

Taken together, these mechanisms suggest that the relationship between social media use and eating behaviors can be conceptualized as a sequential process involving cognitive literacy, attitudinal factors, and emotional regulation. Accordingly, the present study proposes a serial multiple mediation model (PROCESS Model 6), in which the relationship between social media use and eating behaviors is sequentially mediated by e-healthy diet literacy, attitudes toward functional foods, acceptance of food cravings, and emotional eating.

Within this model, social media platforms are conceptualized as complex environments of observational learning, where influencers, peers, and digital content creators function as symbolic role models. Individuals may internalize dietary patterns by repeatedly encountering food-related content across digital platforms (7, 81). Thus, social media is treated not as a singular influence but as a dynamic system through which multiple modeled behaviors are associated with eating-related tendencies.

The integration of SCT, TPB, and SEM provides a comprehensive theoretical framework for understanding these processes. While SCT emphasizes observational learning and environmental exposure, TPB explains how attitudes and norms are associated with behavioral intentions, and SEM situates these processes within broader multi-level contexts. Together, these frameworks enable a more comprehensive understanding of how external influences and internal decision-making processes are jointly associated with eating behaviors.

### 1.9 Conceptual model and mediation pathways

In this study, the conceptual model posits that social media exposure is associated with eating attitudes through four sequential mediators:

- E-Healthy Diet Literacy (M1)
- Attitudes toward Functional Foods (M2)
- Food Craving Acceptance and Behavior (M3)
- Emotional Eating (M4)

This serial mediation structure reflects a cognitive, attitudinal, behavioral, and emotional pathway, providing a theoretically grounded explanation of how digital environments are associated with eating attitudes.

The model proposes the following process:

- Social media exposure is significantly associated with individuals’ digital nutrition literacy.
- Digital nutrition literacy is positively associated with attitudes toward functional foods and health-promoting diets.
- These attitudes, in turn, are associated with the acceptance of food cravings and eating behaviors.
- The regulation of food cravings is associated with emotional eating patterns.
- Emotional eating is subsequently associated with overall eating attitudes.
- Thus, the proposed model represents a multi-stage behavioral mechanism linking digital exposure to dietary attitudes.

### 1.10 Hypotheses development

#### 1.10.1 Social media and E-healthy diet literacy

Social media platforms have become major sources of nutrition-related information, enabling users to access dietary recommendations, recipes, and health advice. However, the quality and reliability of such information vary substantially. Individuals with higher levels of digital diet literacy are better able to critically evaluate online nutritional information and translate it into healthier dietary practices (76).

*H*1: Social media exposure is significantly associated with e-healthy diet literacy.

#### 1.10.2 E-healthy diet literacy and attitudes toward functional foods

Nutrition literacy enhances individuals’ awareness of the health benefits of specific food categories, including functional foods such as probiotic products, fortified foods, and nutraceuticals. Individuals with higher levels of nutrition literacy tend to exhibit more favorable attitudes toward functional foods and health-promoting dietary practices.

*H*2: E-healthy diet literacy is positively associated with attitudes toward functional foods.

#### 1.10.3 Functional food attitudes and food craving acceptance

Attitudes toward functional foods are expected to be associated with how individuals manage food cravings and dietary impulses. Individuals holding positive beliefs about health-promoting foods may demonstrate greater capacity to regulate cravings and engage in healthier eating behaviors.

*H*3: Attitudes toward functional foods are significantly associated with food craving acceptance and eating behavior.

#### 1.10.4 Food craving, acceptance, and emotional eating

Food craving acceptance reflects an individual’s capacity to manage food-related desires without engaging in impulsive eating behaviors. Poor regulation of cravings may increase susceptibility to emotional eating, in which individuals consume food in response to stress or negative emotions.

*H*4: Food craving acceptance is significantly associated with emotional eating.

#### 1.10.5 Emotional eating and eating attitudes

Emotional eating has been consistently associated with maladaptive dietary patterns and negative eating attitudes. Individuals who frequently engage in emotional eating tend to develop dysfunctional relationships with food and exhibit distorted eating attitudes.

*H*5: Emotional eating is significantly associated with eating attitudes.

#### 1.10.6 Direct effects of social media

In addition to indirect pathways, social media exposure may also be directly associated with eating attitudes through mechanisms such as body image internalization, exposure to dietary trends, and peer influence.

*H*6: Social media exposure is significantly associated with eating attitudes.

#### 1.10.7 Serial mediation effects

The proposed model further assumes sequential mediation, in which social media exposure is associated with eating attitudes via a chain of mediators.

*H*7: E-healthy diet literacy mediates the association between social media exposure and eating attitudes.

*H*8: Attitudes toward functional foods mediate the association between social media exposure and eating attitudes.

*H*9: Food craving acceptance and behavior mediate the association between social media exposure and eating attitudes.

*H*10: Emotional eating mediates the association between social media exposure and eating attitudes.

*H*11: Social media exposure is associated with eating attitudes through the sequential mediation of e-healthy diet literacy → functional food attitudes → food craving acceptance → emotional eating.

## 2 Method

### 2.1 Research model

This study was designed within a serial multiple mediation framework to explain the relationship between social media use and eating behaviors. In the proposed model, social media use was specified as the independent variable and eating behavior as the dependent variable.

The model examined the sequential mediating roles of digital healthy eating literacy, attitudes toward functional foods, food craving acceptance, and emotional eating, aiming to clarify the cognitive, attitudinal, and psychological pathways linking social media use to eating behaviors.

The research model is grounded in Social Cognitive Theory and the Theory of Planned Behavior. Within this framework, social media is conceptualized as an environmental factor influencing eating behaviors through observational learning, while attitudes and cognitive evaluations play a key role in behavioral formation (5, 47).

Accordingly, the model assumes that social media use is associated with eating behaviors both directly and indirectly through a sequence of cognitive, attitudinal, and psychological processes. By integrating these pathways, the study adopts a holistic, process-oriented approach that captures the complexity of relationships among key constructs (82, 83).

The model was tested using the PROCESS macro (Model 6), designed for serial multiple mediation analysis. The significance of indirect effects was evaluated using a bootstrap procedure with 5,000 samples, generating bias-corrected confidence intervals for robust estimation (82).

### 2.2 Study model

The proposed study model delineates a sequential mechanism through which social media use influences eating behaviors. Specifically, the model tests the following process chain:

- Social media use → Digital healthy eating literacy.
- Digital healthy eating literacy → Attitudes toward functional foods.
- Attitudes toward functional foods → Acceptance of food cravings
  Acceptance of food cravings → Emotional eating.
- Emotional eating → Eating behavior.
- This sequential structure represents a progression from cognitive processes (digital healthy eating literacy) to attitudinal orientations (attitudes toward functional foods), followed by psychological regulation mechanisms (acceptance of food cravings and emotional eating), ultimately culminating in behavioral outcomes (eating behavior). Accordingly, the model reflects a comprehensive cognitive → attitudinal → psychological → behavioral pathway, offering a theoretically grounded and integrative explanation of how social media use translates into eating-related behaviors.

Digital nutritional literacy represents individuals’ capacity to access, evaluate, and utilize nutrition-related information in online environments and plays a critical role in the development of health-promoting behaviors (31, 39). This cognitive competency can shape behavioral intentions by influencing individuals’ attitudes toward functional foods, thereby linking knowledge-based processes to evaluative orientations (43). In the transition from attitudes to behavior, the way individuals regulate their internal urges becomes particularly important. Acceptance-based approaches facilitate more adaptive behavioral patterns by encouraging individuals to acknowledge and manage their impulses rather than suppress them, thereby enhancing psychological flexibility and self-regulation (17, 84).

Finally, emotional eating, defined as the tendency to consume food in response to negative emotions, emerges as a key determinant of maladaptive eating behaviors (63, 64). Within this framework, emotional regulation processes play a pivotal role in shaping whether individuals’ intentions are translated into healthy or unhealthy dietary outcomes.

### 2.3 Determination of sample size

The required sample size for the present study was determined using a Monte Carlo simulation approach, which is widely recommended for complex mediation models involving multiple indirect pathways. Unlike conventional power analysis methods that primarily focus on direct effects, Monte Carlo simulation enables the estimation of statistical power for indirect and serial indirect effects, which constituted the main analytical focus of this study. The proposed analytical framework corresponded to PROCESS Macro Model 6. It included one independent variable (social media use), four sequential mediators (digital healthy nutrition literacy, attitudes toward functional foods, acceptance of food cravings, and emotional eating), and one dependent variable (eating-related behaviors). Based on prior empirical research on social media use, nutrition literacy, acceptance-based regulation, and emotional eating, small to moderate standardized path coefficients were assumed for the simulation (*β* = 0.20 to 0.30), consistent with effect sizes commonly reported in behavioral and health psychology research.

The Monte Carlo power analysis was conducted in accordance with established methodological procedures. Simulation parameters were specified as follows: standardized path coefficients of *β* = 0.25\beta = 0.25β = 0.25, 10,000 replications, a 95% confidence interval, 5,000 bootstrap resamples, a desired statistical power of 0.80, and a significance level of *α* = 0.05\alpha = 0.05α = 0.05. The primary criterion for adequate power was the detection of the total serial indirect effect. The simulation results indicated that a minimum sample size of approximately *N* = 360 participants was required to achieve 80% power for detecting the full serial indirect effect under the specified assumptions. To account for potential data loss due to incomplete responses, outliers, or case exclusions during data screening, an oversampling strategy was adopted. Accordingly, the target sample size was set at *N* = 400–420 participants.

This sample size is consistent with methodological recommendations for mediation models involving multiple mediators and bootstrapped indirect effects. Overall, Monte Carlo-based estimation provides a more precise and robust basis for sample size determination than traditional rule-of-thumb approaches, particularly in complex psychological models where indirect effects are expected to be relatively small.

### 2.4 Participants

The participants in this study were 1,576 undergraduate students enrolled in the Faculties of Sport Sciences at 38 public universities across Türkiye during the 2025–2026 academic year. A multi-institutional sampling strategy was employed to enhance regional diversity and institutional representativeness. Participants were recruited through convenience sampling, and participation was voluntary. Informed consent was obtained prior to data collection, and anonymity and confidentiality were ensured throughout the study.

The selection of sports science students is both theoretically and practically justified. These individuals are more likely to engage with nutrition-related information due to their academic training and are also potential future professionals who may influence public health behaviors. Their combined exposure to health education and social media environments makes them a particularly relevant population for examining the association between digital influences and eating behaviors (85).

To be included in the study, participants were required to meet the following criteria:

(a) being currently enrolled as an undergraduate student in a sport science-related department (Physical Education and Sport, Coaching Education, Sport Management, or Recreation);
(b) being 18 years of age or older;
(c) providing informed consent to participate; and
(d) completing all measurement instruments in full without missing responses.

Participants were excluded if they:

(a) were not enrolled in a sports science program;
(b) declined to provide informed consent;
(c) submitted incomplete or inconsistent questionnaire responses; or
(d) exhibited response patterns indicative of random or inattentive answering (e.g., identical responses across all items).

After applying the inclusion and exclusion criteria, all 1,576 questionnaires were deemed valid and retained for analysis. The sample size was considered sufficient for testing the proposed serial mediation model. This adequacy was supported by a Monte Carlo–based power analysis, which indicated that the sample size exceeded the minimum required to detect small to moderate indirect effects. Descriptive characteristics of the participants are presented in Table 1.

**Table 1:** Descriptive information of participants.

Variables	Groups	n	%
Gender	Female	765	48.5
	Male	811	51.5
Age	18–22	1,174	74.5
	23–27	247	15.7
	28 and above	155	9.8
Department	Physical education and sport	378	24.0
	Coaching education	395	25.1
	Sport menagement	451	28.6
	Recreation expertation	352	22.3
Academic achievement	0–1.80	102	6.5
	1.81–3.50	1,289	81.8
	3.51–4.00	185	11.7
Weekly studying period	1–10	1,052	66.8
	11–20	401	25.4
	21 and above	123	7.8

Despite the large, geographically diverse sample, participants were exclusively sports science students. This group may differ from the general population in terms of higher physical activity levels, greater health awareness, and structured exposure to nutrition-related education, which may systematically shape both eating behaviors and responses to social media content.

Accordingly, the generalizability of the findings should be interpreted with caution. Rather than representing the general population, sports science students may be considered a health-oriented subgroup with specific behavioral and cognitive profiles. Future research should replicate the model across more diverse populations to enhance external validity (86).

Table 1 presents the descriptive characteristics of the participants. Of the sports science students included in the study, 48.5% were female, and 51.5% were male. In terms of academic specialization, 24.0% were enrolled in Physical Education and Sport, 25.1% in Coaching Education, 28.6% in Sport Management, and 22.3% in Recreation. Regarding academic achievement, 6.5% of participants reported a grade point average (GPA) between 0.00 and 1.80, 81.8% between 1.81 and 3.50, and 11.7% between 3.51 and 4.00. Regarding weekly study time, the majority of students (66.8%) reported studying 1–10 h per week, followed by 25.4% who studied 11–20 h, and 7.8% who studied 21 + hours. (GPA values were based on the Turkish higher education grading system, which ranges from 0.00 to 4.00.)

### 2.5 Data collection tools

In this study, data were collected using the Demographic Information Form, the Social Media’s Effects on Eating Behavior Scale, the e-Healthy Eating Literacy Scale, the Attitudes Toward Functional Foods Scale, the Eating Desire Acceptance and Behavior Scale, the Emotional Eating Scale, and the Eating Attitudes Test Short Form. This multidimensional framework enabled the assessment of eating behavior alongside its underlying cognitive, attitudinal, and psychological processes.

Specifically, the Social Media Effects on Eating Behavior Scale assessed the association between social media use and eating behaviors. The e-Healthy Eating Literacy Scale measured individuals’ ability to access, evaluate, and use digital nutrition information. At the same time, the Attitudes Toward Functional Foods Scale captured awareness of and attitudes toward functional foods.

The Eating Desire Acceptance and Behavior Scale evaluated individuals’ acceptance and regulation of food-related urges, and the Emotional Eating Scale assessed the influence of emotional states on eating behavior. Finally, the Eating Attitudes Test Short Form provided an overall assessment of eating attitudes and potential risk of disordered eating.

The combined use of these instruments enables a comprehensive evaluation of eating behavior by capturing not only observable outcomes but also underlying cognitive, emotional, and attitudinal dimensions. This integrative approach facilitates a more nuanced understanding of the mechanisms shaping eating behavior, particularly among university students, whose dietary patterns are influenced by the interaction of individual, social, digital, and psychological factors.

#### 2.5.1 Social media effects on eating behavior scale

The Social Media Effects on Eating Behavior Scale, developed by Keser, Bayındır Gümüş, Kutlu, and Öztürk (87), is a unidimensional measurement instrument consisting of 18 items. The scale is rated on a five-point Likert scale ranging from “never” (1) to “always” (5). The instrument does not include any reverse-coded items. The total score is calculated by summing all item responses, yielding a possible range of 18–90, with higher scores indicating a greater influence of social media on individuals’ eating behaviors. The scale has demonstrated high internal consistency, with a Cronbach’s alpha coefficient of 0.92, indicating excellent reliability.

#### 2.5.2 E-healthy nutrition literacy scale

The e-Healthy Nutrition Literacy Scale, originally developed by Van Duong et al. (37), has been adapted into Turkish, and its psychometric properties have been validated. The scale is a multidimensional instrument designed to assess individuals’ ability to access, understand, evaluate, and apply information about healthy nutrition in digital environments. The instrument consists of 15 items distributed across five sub-dimensions: finding, understanding, evaluating, and applying e-healthy nutrition information, as well as digital healthy nutrition literacy. All items are rated on a five-point Likert-type scale. The Turkish validity and reliability study was conducted by Onbaşı and Türker (88). Confirmatory factor analysis results (χ^2^/df = 4.25; AGFI = 0.91; RMSEA = 0.068) indicate that the five-factor structure demonstrates an acceptable level of model fit. The scale’s internal consistency was reported as Cronbach’s *α* = 0.77, indicating satisfactory reliability. Higher scores obtained from the scale reflect higher levels of digital healthy nutrition literacy among individuals.

#### 2.5.3 Attitudes toward functional foods scale

The Attitudes Toward Functional Foods Scale, developed by Hacıoğlu and Kurt (89), was used to assess individuals’ awareness, acceptance, and overall attitudes toward functional foods. The scale is designed to evaluate consumers’ perceptions of foods that provide health benefits beyond basic nutritional value. The instrument consists of multiple items rated on a five-point Likert scale, ranging from “strongly disagree” (1) to “strongly agree” (5). Higher scores indicate more positive attitudes toward functional foods. Factor analysis results indicate that the scale comprises four sub-dimensions: utility, necessity, trust, and safety. Reliability analyses showed that Cronbach’s alpha coefficients for these sub-dimensions ranged from 0.794 to 0.901, indicating high internal consistency.

#### 2.5.4 Food craving acceptance and action questionnaire

The Food Craving Acceptance and Action Questionnaire (FAAQ), originally developed by Juarascio et al. (90), was used to assess individuals’ levels of acceptance and psychological flexibility in response to food cravings. The scale is grounded in acceptance-based theoretical frameworks and evaluates how individuals respond to food-related urges. The Turkish adaptation of the scale was conducted by Hamurcu et al. (91). The instrument consists of 10 items organized into two sub-dimensions and is rated on a five-point Likert scale ranging from “strongly disagree” (1) to “strongly agree” (5). The scale does not include any reverse-coded items. Total scores range from 10 to 50, with higher scores indicating greater acceptance and psychological flexibility in managing food cravings. The Turkish validity and reliability study confirmed the scale’s two-factor structure and reported a high level of internal consistency (Cronbach’s *α* = 0.92), indicating excellent reliability (91).

#### 2.5.5 Emotional eating scale

The Emotional Eating Scale (EES), developed by Garaulet et al. (92), was used to assess individuals’ tendency to engage in eating behavior in response to emotional states. The Turkish adaptation of the scale was conducted by Arslantaş et al. (93). The scale consists of 10 items organized into three subdimensions: inability to control cravings, types of food, and feelings of guilt. Items are rated on a four-point Likert scale ranging from “never” (0) to “always” (3). The scale does not include any reverse-coded items. Total scores range from 0 to 30, with higher scores indicating greater levels of emotional eating. The Turkish version of the scale demonstrated satisfactory internal consistency, with a Cronbach’s alpha coefficient of 0.84 (93).

#### 2.5.6 Eating attitudes test–short form (EAT-26)

The Eating Attitudes Test–Short Form (EAT-26) is a 26-item self-report instrument derived from the original Eating Attitudes Test (EAT-40), developed by Garner and Garfinkel (94) and subsequently shortened by Garner et al. (95). The Turkish validity and reliability of the scale were established by Ergüney Okumuş and Sertel Berk (96). The scale consists of 26 items rated on a 6-point Likert scale, with response options “always,” “very often,” “frequently,” “sometimes,” “rarely,” and “never.” Scoring is based on standard procedures, whereby responses of “always,” “very often,” and “frequently” are assigned scores of 3, 2, and 1, respectively, while the remaining responses are scored as 0. Item 26 is reverse-coded, with “sometimes,” “rarely,” and “never” assigned scores of 1, 2, and 3, respectively. The EAT-26 demonstrates a three-factor structure consisting of dieting, bulimia, and preoccupation with eating/control. Higher total scores indicate more problematic eating attitudes and a greater risk of eating disorders. A total score of 20 or above is commonly used as a cutoff point, indicating elevated risk for disordered eating behaviors.

Table 2 indicates that all measurement instruments used in the study demonstrate high levels of internal consistency. Cronbach’s alpha coefficients ranged from 0.85 to 0.94, reflecting strong reliability across the scales. Overall, these findings provide robust evidence that the instruments employed in the study yield consistent and reliable measurements, supporting their suitability for subsequent analyses.

**Table 2:** Descriptive values of scales.

Scales	Number of items	Cronbach’s alpha
Social media on eating behavior	18	0.94
E-healthy diet literacy	15	0.86
Attitudes toward functional foods	25	0.92
Food carving acceptance and action	10	0.87
Emotional eater	10	0.85
Eating attitudes	26	0.94

Table 3 presents the confirmatory factor analysis (CFA) model fit indices for the six measurement scales used in the study. The modeThThe results of the confirmatory factor analysis (CFA) indicated that all measurement models demonstrated acceptable to excellent fit with the data. The χ^2^/df ratios for the six scales ranged from 2.18 to 2.98, remaining below the recommended threshold of 3, indicating a good model fit. Similarly, the Comparative Fit Index (CFI) ranged from 0.945 to 0.997, and the Tucker–Lewis Index (TLI) ranged from 0.913 to 0.976, both exceeding the recommended cutoff of 0.90, suggesting satisfactory model fit across all constructs.

**Table 3:** Confirmatory factor analysis (CFA) model fit indices.

Fit index	Recommended value	Social media and eating behavior	Digital healthy diet literacy	Functional food attitude	Food craving acceptance	Emotional eating	Eating attitudes
χ^2^/df	< 3	2.428	2.98	2.18	2.97	2.295	2.977
CFI	≥ 0.90	0.997	0.945	0.994	0.951	0.985	0.957
TLI	≥ 0.90	0.967	0.927	958	0.913	0.976	0.939
RMSEA	≤ 0.08	0.047	0.057	0.046	0.078	0.038	0.075
SRMR	≤ 0.08	0.046	0.043	0.040	0.048	0.019	0.043
GFI	≥ 0.90	0.995	0.959	0.968	0.963	0.988	0.966
AGFI	≥ 0.85	0.948	0.939	0.996	0.919	0.977	0.937

The Root Mean Square Error of Approximation (RMSEA) values varied between 0.038 and 0.078, all remaining below the acceptable limit of 0.08, indicating an adequate approximation of the population covariance matrix. Additionally, the Standardized Root Mean Square Residual (SRMR) values ranged from 0.019 to 0.048, well below the recommended threshold of 0.08, further supporting the adequacy of the models. Goodness-of-Fit Index (GFI) values (0.959–0.995) and Adjusted Goodness-of-Fit Index (AGFI) values (0.919–0.977) also exceeded the acceptable limits of 0.90 and 0.85, respectively. Overall, these findings indicate that the measurement models for Social Media and Eating Behavior, Digital Healthy Diet Literacy, Functional Food Attitude, Food Craving Acceptance, Emotional Eating, and Eating Attitudes demonstrate satisfactory structural validity and an overall good model fit.

### 2.6 Measures

#### 2.6.1 Discriminant validity assessment

Discriminant validity was assessed using the Fornell–Larcker criterion, which requires that the square root of the Average Variance Extracted (AVE) for each construct exceeds its correlations with other constructs (97).

The results showed that the square roots of the AVEs for all constructs were higher than the corresponding inter-construct correlations, indicating that each construct is empirically distinct and captures a unique aspect of the model. These findings provide evidence of adequate discriminant validity, suggesting that the measurement model meets established criteria for construct distinctiveness (98).

However, several constructs exhibited moderate-to-high intercorrelations (r ≥ 0.60), suggesting partial conceptual overlap among mediators. Such patterns are not uncommon in complex psychological models, particularly when constructs represent related cognitive and affective processes (99). This suggests that the mediators may reflect interconnected dimensions of a broader underlying mechanism rather than entirely independent constructs.

Accordingly, the serial mediation structure should be interpreted as a theoretically guided representation of related processes. Future research employing alternative approaches, such as longitudinal designs or bifactor modeling, may further clarify construct distinctiveness.

#### 2.6.2 Robustness analysis using knowledge dimension

To further examine the stability and robustness of the findings, an additional analysis was conducted by incorporating a knowledge-related control variable into the model. Specifically, the knowledge dimension (e.g., domain-specific educational background or training exposure) was included as a control variable in the regression analysis. Previous research has shown that knowledge and educational exposure can influence behavioral tendencies, cognitive processing, and decision-making patterns (100).

The results indicated that including the knowledge dimension variable did not substantially alter the magnitude, direction, or statistical significance of the model’s primary relationships. This suggests that the observed associations are stable and not driven by differences in participants’ underlying knowledge. Consequently, the findings support the robustness of the proposed model and reinforce the validity of the study’s conclusions.

#### 2.6.3 Common method bias

Given that all variables were collected using self-report measures, common method bias (CMB) was assessed. Harman’s single-factor test was conducted, and the results indicated that the first factor accounted for less than 50% of the total variance, suggesting that common method bias was not a serious concern.

Additionally, a full collinearity assessment approach was applied, and variance inflation factor (VIF) values for all constructs were below the recommended threshold of 3.3, further indicating the absence of significant common method bias (101).

#### 2.6.4 Analysis of data

Data was analyzed using SPSS v22. The Kolmogorov–Smirnov test, one of the tests used to assess the normality of data distributions (102), was employed. The normality results of the scores obtained in this study are presented in Table 4.

**Table 4:** Skewness, Kurtosis, and Kolmogorov–Smirnov test significance level results of the participants’ scale scores.

Scales	Skewness	Kurtosis
Social media on eating behavior	0.06	−0.20
E-healthy diet literacy	−0.19	0.88
Attitudes toward functional foods	−0.23	2.12
Food carving acceptance and action	0.07	0.98
Emotional eater	−0.13	0.51
Eating attitudes	0.15	0.66

When Table 4 is examined, skewness and kurtosis values fall within ±2, indicating no substantial deviation from normality and supporting the use of parametric tests. Pearson correlation analysis was conducted to examine relationships among variables, and regression analysis was used to assess associations between social media–related factors, eating behavior, and eating attitudes.

To test the proposed serial mediation model, regression-based indirect effect analysis was performed using the PROCESS Macro Model 6 (103) with 5,000 bootstrap resamples. Indirect effects were considered statistically significant when the 95% confidence intervals did not include zero (103, 104).

In addition, a Common Latent Factor (CLF) test was conducted to assess Common Method Bias (CMB). The inclusion of the CLF did not result in substantial changes in standardized coefficients (*Δ* < 0.20), suggesting that common method variance was not a serious concern (105).

Although the model was theoretically grounded in Social Cognitive Theory and the Theory of Planned Behavior, the cross-sectional design limits causal inference and temporal ordering. Accordingly, the specified pathways should be interpreted as theoretically informed associations rather than causal effects (106). While serial mediation with bootstrapping allows examination of indirect mechanisms (82), future longitudinal and experimental studies are needed to establish causality.

## 3 Results

Table 5 presents the mean and standard deviation values of the study variables among sports science students. The results indicate that participants had an average score of 46.90 ± 15.84 for Social Media Effects on Eating Behavior, 38.50 ± 8.94 for e-Healthy Diet Literacy, 75.18 ± 16.92 for Attitudes toward Functional Foods, 35.51 ± 8.47 for Food Craving Acceptance and Action, 25.64 ± 6.10 for Emotional Eating, and 83.70 ± 24.22 for Eating Attitudes.

**Table 5:** Descriptive statistics of variables.

Scales	n	Min.	Max.	M ± SD
Social media on eating behavior	1,576	18.00	90.00	46.90 ± 15.84
E-healthy diet literacy		15.00	63.00	38.50 ± 8.94
Attitudes toward functional foods		25.00	125.00	75.18 ± 16.92
Food carving acceptance and action		10.00	60.00	35.51 ± 8.47
Emotional eater		10.00	40.00	25.64 ± 6.10
Eating attitudes		26.00	156.00	83.70 ± 24.22

Table 6 presents the correlation coefficients among the study variables. The results indicate that Social Media and Eating Behavior were positively and significantly correlated with e-Healthy Diet Literacy (*r* = 0.55), Attitudes Toward Functional Foods (*r* = 0.54), Food Craving Acceptance and Action (*r* = 0.24), Emotional Eating (*r* = 0.53), and Eating Attitudes (*r* = 0.55; *p* < 0.01).

**Table 6:** Pearson correlation coefficients for the relationships between variables.

Scales	1	2	3	4	5	6
1-Social media on eating behavior	1	0.55**	0.54**	0.24**	0.53**	0.55**
2-E-healthy diet literacy		1	0.62**	0.49**	0.47**	0.52**
3-Attitudes toward functional foods			1	0.60**	0.54**	0.57**
4-Food carving acceptance and action				1	0.36**	0.42**
5-Emotional eater					1	0.61**
6-Eating attitudes						1

**p < 0.01, *n* = 1,576, 1-Social Media on Eating Behavior, 2-E-Healthy Diet Literacy, 3-Attitudes Toward Functional Foods, 4-Food Carving Acceptance and Action, 5-Emotional Eater, 6-Eating Attitudes.

Similarly, e-Healthy Diet Literacy was positively and significantly associated with Attitudes Toward Functional Foods (*r* = 0.62), Food Craving Acceptance and Action (*r* = 0.49), Emotional Eating (*r* = 0.47), and Eating Attitudes (*r* = 0.52; *p* < 0.01). Attitudes Toward Functional Foods also showed positive and significant correlations with Food Craving Acceptance and Action (*r* = 0.60), Emotional Eating (*r* = 0.54), and Eating Attitudes (*r* = 0.57; *p* < 0.01).

Food Craving Acceptance and Action was positively and significantly correlated with Emotional Eating (*r* = 0.36) and Eating Attitudes (*r* = 0.42; *p* < 0.01). Finally, a strong positive and significant correlation was observed between Emotional Eating and Eating Attitudes (*r* = 0.61; *p* < 0.01).

Interestingly, the positive association between attitudes toward functional foods and acceptance of food cravings may initially appear counterintuitive. From a conventional health-behavior perspective, stronger health-oriented attitudes would be expected to reduce food cravings. However, this finding can be more appropriately interpreted within the framework of acceptance-based self-regulation. Individuals with higher levels of nutrition-related awareness and more favorable attitudes toward functional foods may be more likely to adopt adaptive regulatory strategies, such as acknowledging cravings without acting upon them, rather than engaging in rigid suppression attempts.

This interpretation aligns with acceptance-based models, which suggest that non-judgmental awareness and acceptance of internal experiences can reduce maladaptive behavioral responses and enhance self-regulatory capacity (17, 84). Accordingly, higher levels of food craving acceptance in this context may reflect improved psychological flexibility and more adaptive regulation processes, rather than increased susceptibility to unhealthy eating behaviors.

**Figure 1:**
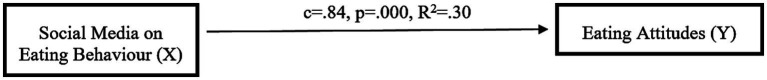
The effect of social media on eating behavior and eating attitudes.

Table 7 presents the results of the regression analysis examining the association between social media and eating behavior and eating attitudes. The findings indicate that social media and eating behavior were positively and significantly associated with Eating Attitudes (*β* = 0.84, SE = 0.03, t = 26.00, *p* < 0.001). The overall regression model was statistically significant (*F* = 676.18, *p* < 0.001) and accounted for 30% of the variance in Eating Attitudes (R^2^ = 0.30). These results suggest that social media–related aspects of eating behavior are strongly associated with individuals’ eating attitudes (see Figure 1).

**Table 7:** The effect of social media on eating behavior and eating attitudes.

Variables		β	*SE*	*t*	*p*	*R*	*R* ^2^	*F*	*p*
Independent	Depend								
Social media on eating behavior	Eating attitudes	0.84	0.03	26.00	0.000	0.55	0.30	676.18	0.000**

**p < 0.01.

Table 8 presents the multicollinearity diagnostics for the independent variables included in the regression model. The results indicate that tolerance values ranged from 0.419 to 0.621, while variance inflation factor (VIF) values ranged from 1.610 to 2.388. According to established criteria, tolerance values greater than 0.20 and VIF values below 10 indicate no multicollinearity concern.

**Table 8:** Collinearity statistics of predictor variables.

Model	Collinearity statistics	
1	Tolerance	VIF
Effects of social media on eating behavior	0.556	1.799
E-healthy nutrition literacy	0.513	1.949
Attitudes toward functional foods	0.419	2.388
Acceptance of food cravings and eating behavior	0.591	1.692
Emotional eating	0.621	1.610

In line with these thresholds, the findings suggest that multicollinearity is not a concern in the present model. Therefore, the independent variables Social Media and Eating Behavior, e-Healthy Eating Literacy, Attitudes Toward Functional Foods, Food Craving Acceptance and Action, and Emotional Eating do not exhibit problematic intercorrelations, and their inclusion in the regression analysis is statistically appropriate.

Table 9 presents the Cook’s Distance values calculated to assess the presence of influential observations in the regression model. The results indicate that the minimum Cook’s Distance value is 0.000, the maximum value is 0.149, and the mean value is 0.001. According to commonly accepted criteria, Cook’s Distance values below 1 indicate the absence of influential outliers.

**Table 9:** Cook’s distance statistics.

Statistic	Min.	Max.	Mean	SD	N
Cook’s distance	0.000	0.149	0.001	0.007	1,576

In line with this threshold, the findings suggest that no extreme or influential observations are present in the dataset. Therefore, it can be concluded that the data do not violate the assumptions regarding influential cases and are suitable for regression analysis.

Table 10 presents the summary statistics for the multiple linear regression model predicting eating attitudes. The results indicate that the overall model is statistically significant (*F* = 315.147, *p* < 0.01). A strong positive relationship was observed between the set of independent variables and the dependent variable (R = 0.708). Examination of the model’s explanatory power revealed that the independent variables accounted for approximately 50.1% of the variance in eating attitudes (R^2^ = 0.501; Adjusted R^2^ = 0.499). This finding suggests that the variables included in the model provide a substantial and meaningful explanation of eating attitudes.

**Table 10:** Regression model summary.

Model	R	R^2^	Adjusted R^2^	Std. error	F	*p*
1	0.708	0.501	0.499	17.13599	315.147	0.000**

**p < 0.01.

Table 11 presents the results of the ANOVA conducted to evaluate the overall significance of the multiple linear regression model. The findings indicate that the model is statistically significant (*F*(5, 1,570) = 315.147, *p* < 0.01). The total sum of squares was calculated as 923,719.837, of which the regression model explained 462,701.873. These results demonstrate that the set of independent variables, when considered collectively, significantly explains the variance in eating attitudes.

**Table 11:** Analysis of variance (ANOVA) for the regression model.

Model		Sum of squares	df	Mean square	F	*p*
1	Regression	462701.873	5	92540.375	315.147	0.000**
	Residual	461017.964	1,570	293.642		
	Total	923719.837	1,575			

**p < 0.01.

Table 12 presents the regression coefficients for the variables associated with eating attitudes. The results indicate that all independent variables included in the model are significantly associated with eating attitudes (*p* < 0.01). Examination of the standardized coefficients shows that emotional eating has the strongest association with eating attitudes (*β* = 0.333, t = 14.723, *p* < 0.01). This is followed by Social Media and Eating Behavior (*β* = 0.213), Attitudes Toward Functional Foods (*β* = 0.143), Food Craving Acceptance and Action (*β* = 0.112), and e-Healthy Eating Literacy (*β* = 0.101).

**Table 12:** Regression coefficients for predicting eating attitudes.

Model	Unstandardized coefficients	Standardized coefficients			
Scales	B	Std. error	β	t	*p*
Effects of social media on eating behavior	0.326	0.037	0.213	8.921	0.000**
E-healthy nutrition literacy	0.273	0.067	0.101	4.047	0.000**
Attitudes toward functional foods	0.205	0.039	0.143	5.196	0.000**
Acceptance of food cravings and eating behavior	0.287	0.059	0.112	4.849	0.000**
Emotional eating	1.322	0.090	0.333	14.723	0.000**

**p < 0.01.

Overall, these findings demonstrate that both cognitive and psychological variables are significantly associated with eating attitudes, with emotional eating emerging as the most prominent factor.

When Table 13 is examined, it is observed that Social Media and Eating Behavior (X) is positively and significantly associated with e-Healthy Eating Literacy (M1) (a₁ = 0.31, SE = 0.01, t = 26.04, *p* < 0.001). This finding indicates that higher levels of social media-related eating behavior are associated with higher levels of digital healthy eating literacy. The model explains 30% of the variance in M1 (R^2^ = 0.30, *F*(1, 1,574) = 677.95, *p* < 0.001).

**Table 13:** The serial mediation role of e-healthy diet literacy, attitudes toward functional foods, food carving acceptance and action, and emotional eater between social media on eating behavior and eating attitudes (*N* = 1,576).

Scales	Outcomes																			
	E-healthy diet literacy (M1)				Attitudes toward functional foods (M2)				Food carving acceptance and action (M3)				Emotional eater (M4)				Eating attitudes (Y)			
		b	SE	t		b	SE	t		b	SE	t		b	SE	t		b	SE	t
Social media on eating behavior (X)	a_1_	0.31	0.01	26.04	a_2_	0.31	0.02	12.78	a3	−0.12	0.02	−7.96	a4	0.12	0.01	12.22	c’	0.33	0.04	8.92
E-healthy diet literacy (M1)					d_21_	0.88	0.04	20.54	d_31_	0.29	0.03	10.26	d_41_	0.06	0.02	3.28	b1	0.27	0.07	4.05
Attidudes toward functional foods (M2)									d_32_	0.30	0.02	20.53	d_42_	0.10	0.01	8.80	b2	0.21	0.04	5.20
Food carving acceptance and action (M3)													d_43_	0.06	0.02	3.30	b3	0.29	0.06	4.85
Emotional eater (M4)																	b4	1.32	0.09	14.72
Constant																				
		R2 = 0.30				R2 = 0.44				R2 = 0.41				R2 = 0.38				R2 = 0.50		
		*F*(1,1,574) = 677.95				F(1,1,574) = 624.80				F(1,1,574) = 356.32				F(1,1,574) = 239.72				F(1,1,574) = 315.15		
		*p* = 0.000**				*p* = 0.000**				*p* = 0.000**				*p* = 0.000**				*p* = 0.000**		

**p < 0.01.

Similarly, Social Media and Eating Behavior are positively and significantly associated with Attitudes Toward Functional Foods (M2; a₂ = 0.31, SE = 0.02, t = 12.78, *p* < 0.001), explaining 44% of the variance (R^2^ = 0.44, F(1, 1,574) = 624.80, *p* < 0.001). This result suggests that greater social media exposure is associated with more favorable attitudes toward functional foods.

In contrast, Social Media and Eating Behavior is negatively and significantly associated with Food Craving Acceptance and Action (M3; a₃ = −0.12, SE = 0.02, t = −7.96, *p* < 0.001), indicating that increased social media-related eating behavior is associated with lower levels of acceptance and psychological flexibility regarding food cravings. The model explains 41% of the variance in M3 (R^2^ = 0.41, F(1, 1,574) = 356.32, *p* < 0.001).

Furthermore, Social Media and Eating Behavior is positively and significantly associated with Emotional Eating (M4; a₄ = 0.12, SE = 0.01, t = 12.22, *p* < 0.001), accounting for 38% of the variance (R^2^ = 0.38, F(1, 1,574) = 239.72, *p* < 0.001). This finding indicates that higher social media exposure is associated with higher levels of emotional eating.

Regarding inter-mediator relationships, e-Healthy Eating Literacy (M1) is positively and significantly associated with Attitudes Toward Functional Foods (M2; d₂₁ = 0.88, SE = 0.04, t = 20.54, *p* < 0.001). It is also significantly associated with Food Craving Acceptance and Action (M3; d₃₁ = 0.29, SE = 0.03, t = 10.26, *p* < 0.001) and Emotional Eating (M4; d₄₁ = 0.06, SE = 0.02, t = 3.28, *p* < 0.01).

Attitudes Toward Functional Foods (M2) is significantly associated with Food Craving Acceptance and Action (M3; d₃₂ = 0.30, SE = 0.02, t = 20.53, *p* < 0.001) and Emotional Eating (M4; d₄₂ = 0.10, SE = 0.01, t = 8.80, *p* < 0.001). Additionally, Food Craving Acceptance and Action (M3) is significantly associated with Emotional Eating (M4; d₄₃ = 0.06, SE = 0.02, t = 3.30, *p* < 0.01).

In addition, all mediating variables are significantly associated with Eating Attitudes (Y): e-Healthy Eating Literacy (b₁ = 0.27, SE = 0.07, t = 4.05, *p* < 0.001), Attitudes Toward Functional Foods (b₂ = 0.21, SE = 0.04, t = 5.20, *p* < 0.001), Food Craving Acceptance and Action (b₃ = 0.29, SE = 0.06, t = 4.85, *p* < 0.001), and Emotional Eating (b₄ = 1.32, SE = 0.09, t = 14.72, *p* < 0.001).

Finally, the direct asThe association of Social Media and Eating Behavior with Eating Attitudes remains positive and statistically significant when controlling for the mediators (c′ = 0.33, SE = 0.04, t = 8.92, *p* < 0.001), indicating partial mediation. The model predicting Eating Attitudes explains approximately 50% of the total variance (R^2^ = 0.50, *F*(5, 1,574) = 315.15, *p* < 0.001), indicating substantial explanatory power.

These findings suggest that eating attitudes are associated with both direct and indirect pathways, supporting the proposed serial mediation structure of the model. Although the indirect effects were statistically significant, the full serial mediation effect was relatively small. This finding is not unexpected given the large sample size (*N* = 1,576), which increases statistical power and the likelihood of detecting small effects (107).

From a practical perspective, the results suggest that while the proposed mechanisms are statistically reliable, their real-world impact may be modest. Therefore, the findings should be interpreted as contributing to theoretical understanding rather than indicating strong practical effects.

Interestingly, the positive association between attitudes toward functional foods and acceptance of food cravings may initially appear counterintuitive. From a conventional health-behavior perspective, stronger health-oriented attitudes would be expected to reduce food cravings. However, this finding can be more appropriately interpreted within the framework of acceptance-based self-regulation. Individuals with higher levels of nutrition-related awareness and more favorable attitudes toward functional foods may be more likely to adopt adaptive regulatory strategies, such as acknowledging cravings without acting upon them, rather than engaging in rigid suppression attempts.

This interpretation aligns with acceptance-based models, which suggest that non-judgmental awareness and acceptance of internal experiences can reduce maladaptive behavioral responses and enhance self-regulatory capacity (17, 84). Accordingly, higher levels of food craving acceptance in this context may reflect improved psychological flexibility and more adaptive regulation processes, rather than increased susceptibility to unhealthy eating behaviors.

**Figure 2:**
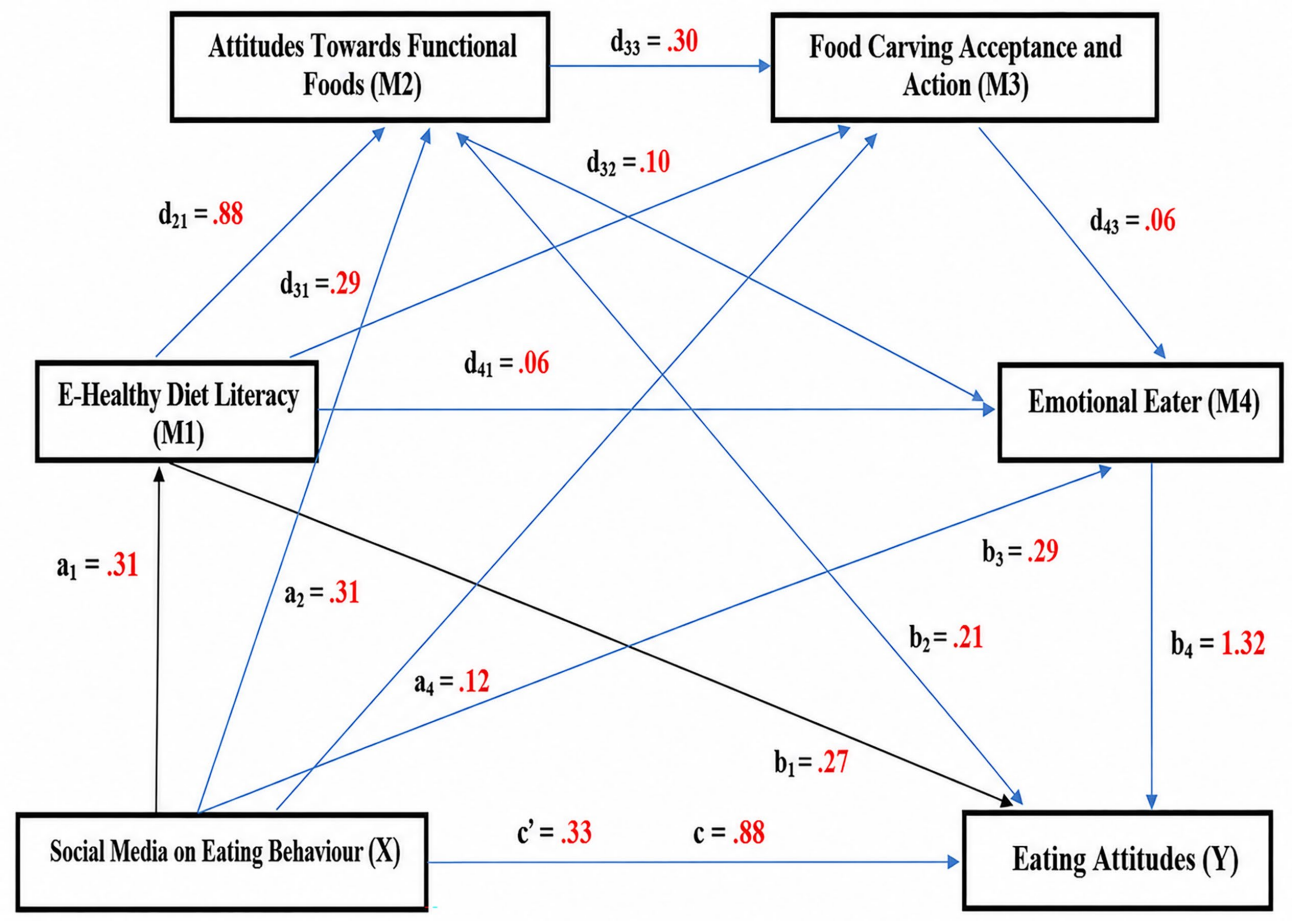
Serial mediation model of social media use and eating attitudes via e-healthy diet literacy, attitudes toward functional foods, food craving acceptance and action, and emotional eating.

As illustrated in Figure 2, a serial multiple mediation model was employed in this study. In this model, Social Media and Eating Behavior (X) was specified as the independent variable, Eating Attitudes (Y) as the dependent variable. Four sequential mediators were included: e-Healthy Eating Literacy (M1), Attitudes Toward Functional Foods (M2), Food Craving Acceptance and Action (M3), and Emotional Eating (M4). Within this framework, indirect effects were examined systematically at multiple levels:

### 3.1 Specific (simple) indirect effects

- Social Media and Eating Behavior → e-Healthy Eating Literacy → Eating Attitudes (a₁b₁).
- Social Media and Eating Behavior → Attitudes Toward Functional Foods → Eating Attitudes (a₂b₂).
- Social Media and Eating Behavior → Food Craving Acceptance and Action → Eating Attitudes (a₃b₃).
- Social Media and Eating Behavior → Emotional Eating → Eating Attitudes (a₄b₄).

### 3.2 Two-step (dual mediator) indirect effects

- Social Media and Eating Behavior → e-Healthy Eating Literacy → Attitudes Toward Functional Foods → Eating Attitudes (a₁d₂₁b₂).
- Social Media and Eating Behavior → e-Healthy Eating Literacy → Food Craving Acceptance and Action → Eating Attitudes (a₁d₃₁b₃).
- Social Media and Eating Behavior → e-Healthy Eating Literacy → Emotional Eating → Eating Attitudes (a₁d₄₁b₄).
- Social Media and Eating Behavior → Attitudes Toward Functional Foods → Food Craving Acceptance and Action → Eating Attitudes (a₂d₃₂b₃).
- Social Media and Eating Behavior → Attitudes Toward Functional Foods → Emotional Eating → Eating Attitudes (a₂d₄₂b₄).
- Social Media and Eating Behavior → Food Craving Acceptance and Action → Emotional Eating → Eating Attitudes (a₃d₄₃b₄).

### 3.3 Higher order (multiple-step) indirect effects

- Social Media and Eating Behavior → e-Healthy Eating Literacy → Attitudes Toward Functional Foods → Food Craving Acceptance and Action → Eating Attitudes (a₁d₂₁d₃₂b₃).
- Social Media and Eating Behavior → e-Healthy Eating Literacy → Attitudes Toward Functional Foods → Emotional Eating → Eating Attitudes (a₁d₂₁d₄₂b₄).
- Social Media and Eating Behavior → e-Healthy Eating Literacy → Food Craving Acceptance and Action → Emotional Eating → Eating Attitudes (a₁d₃₁d₄₃b₄).
- Social Media and Eating Behavior → e-Healthy Eating Literacy → Attitudes Toward Functional Foods → Food Craving Acceptance and Action → Emotional Eating → Eating Attitudes (a₁d₂₁d₃₂d₄₃b₄).

Each of these pathways illustrates how the association between the independent and dependent variables is transmitted through different combinations of mediators. The sum of all specific indirect effects constitutes the total indirect (serial mediation) association of Social Media and Eating Behavior with Eating Attitudes. In addition, the direct effect (c′) included in the model represents the association between Social Media and Eating Behavior and Eating Attitudes after controlling for all mediating variables.

As presented in Table 14, the total indirect association of Social Media and Eating Behavior with Eating Attitudes was positive and statistically significant (B = 0.51, SE = 0.04, 95% CI [0.44, 0.58]). This finding indicates that the overall mediating structure significantly links social media–related eating behavior with eating attitudes.

**Table 14:** The indirect effects of social media on eating behavior and eating attitudes (*n* = 1,576).

Indirect effects		b	SE	LLCI	ULCI
Total		0.51	0.04	0.44	0.58
Ind 1	Social Media on Eating Behavior→E-Healthy Diet Literacy →Eating Attitudes	0.09	0.03	0.03	0.14
Ind 2	Social Media on Eating Behavior→Attidudes Toward Functional Foods→Eating Attitudes	0.06	0.02	0.02	0.11
Ind 3	Social Media on Eating Behavior →Food Carving Acceptance and Action→Eating Attitudes	−0.03	0.01	−0.06	−0.01
Ind 4	Social Media on Eating Behavior→Emotional Eater→Eating Attitudes	0.16	0.02	0.12	0.20
Ind 5	Social Media on Eating Behavior→E-Healthy Diet Literacy→Attitudes Toward Functional Foods → Eating Attitudes	0.06	0.02	0.02	0.10
Ind 6	Social Media on Eating Behavior→E-Healthy Diet Literacy→Food Carving Acceptance and Action →Eating Attitudes	0.03	0.01	0.01	0.04
Ind 7	Social Media on Eating Behavior→E-Healthy Diet Literacy→Emotional Eater →Eating Attitudes	0.03	0.01	0.01	0.05
Ind 8	Social Media on Eating Behavior→Attidudes Toward Functional Foods→Food Carving Acceptance and Action→Eating Attitudes	0.03	0.01	0.01	0.05
Ind 9	Social Media on Eating Behavior→Attidudes Toward Functional Foods→Emotional Eater→Eating Attitudes	0.04	0.01	0.02	0.06
Ind 10	Social Media on Eating Behavior→Food Carving Acceptance and Action→Emotional Eater→Eating Attitudes	−0.01	0.01	−0.02	−0.01
Ind 11	Social Media on Eating Behavior→E-Healthy Diet Literacy→Attitudes Toward Functional Foods →Food Carving Acceptance and Action→Eating Attitudes	0.02	0.01	0.01	0.03
Ind 12	Social Media on Eating Behavior→E-Healthy Diet Literacy→Attitudes Toward Functional Foods →Emotional Eater →Eating Attitudes	0.03	0.01	0.02	0.05
Ind 13	Social Media on Eating Behavior→E-Healthy Diet Literacy→Food Carving Acceptance and Action → Emotional Eater→Eating Attitudes	0.01	0.01	0.01	0.01
Ind 14	Social Media on Eating Behavior→Attidudes Toward Functional Foods→Food Carving Acceptance and Action→Emotional Eater→Eating Attitudes	0.01	0.01	0.01	0.01
Ind 15	Social Media on Eating Behavior→E-Healthy Diet Literacy→Attitudes Toward Functional Foods →Food Carving Acceptance and Action→Emotional Eater→Eating Attitudes	0.01	0.01	0.01	0.01

The first specific indirect effect, representing the pathway through e-Healthy Eating Literacy (Ind₁: X → M₁ → Y), was positive and statistically significant (B = 0.09, SE = 0.03, 95% CI [0.03, 0.14]), suggesting that higher levels of social media–related eating behavior are associated with increased digital healthy eating literacy, which in turn is associated with eating attitudes.

The second indirect effect, operating through Attitudes Toward Functional Foods (Ind₂: X → M₂ → Y), was also positive and statistically significant (B = 0.06, SE = 0.02, 95% CI [0.02, 0.11]), indicating that social media exposure is associated with more favorable attitudes toward functional foods, which are in turn associated with eating attitudes.

In contrast, the third indirect effect, representing the pathway through Food Craving Acceptance and Action (Ind₃: X → M₃ → Y), was negative and statistically significant (B = −0.03, SE = 0.01, 95% CI [−0.06, −0.01]), suggesting that higher social media–related eating behavior is associated with lower levels of acceptance and psychological flexibility regarding food cravings, which is in turn associated with eating attitudes.

The fourth indirect effect, representing the pathway through Emotional Eating (Ind₄: X → M₄ → Y), was positive and statistically significant (B = 0.16, SE = 0.02, 95% CI [0.12, 0.20]), indicating that social media–related eating behavior is associated with higher emotional eating, which is in turn associated with eating attitudes.

The fifth indirect effect reflects a sequential pathway through e-Healthy Eating Literacy and Attitudes Toward Functional Foods (Ind₅: X → M₁ → M₂ → Y), which was positive and statistically significant (B = 0.06, SE = 0.02, 95% CI [0.02, 0.10]).

The sixth indirect effect, through e-Healthy Eating Literacy and Food Craving Acceptance and Action (Ind₆: X → M₁ → M₃ → Y), was also statistically significant (B = 0.03, SE = 0.01, 95% CI [0.01, 0.04]).

The seventh indirect effect, through e-Healthy Eating Literacy and Emotional Eating (Ind₇: X → M₁ → M₄ → Y), was positive and statistically significant (B = 0.03, SE = 0.01, 95% CI [0.01, 0.05]).

The eighth indirect effect, representing the pathway through Attitudes Toward Functional Foods and Food Craving Acceptance and Action (Ind₈: X → M₂ → M₃ → Y), was positive and statistically significant (B = 0.03, SE = 0.01, 95% CI [0.01, 0.05]).

The ninth indirect effect, through Attitudes Toward Functional Foods and Emotional Eating (Ind₉: X → M₂ → M₄ → Y), was also positive and statistically significant (B = 0.04, SE = 0.01, 95% CI [0.02, 0.06]).

The tenth indirect effect, through Food Craving Acceptance and Action and Emotional Eating (Ind₁₀: X → M₃ → M₄ → Y), was negative and statistically significant (B = −0.01, SE = 0.01, 95% CI [−0.02, −0.01]).

The eleventh indirect effect, representing a higher-order sequential pathway through e-Healthy Eating Literacy, Attitudes Toward Functional Foods, and Food Craving Acceptance and Action (Ind₁₁: X → M₁ → M₂ → M₃ → Y), was positive and statistically significant (B = 0.02, SE = 0.01, 95% CI [0.01, 0.03]).

The twelfth indirect effect, through e-Healthy Eating Literacy, Attitudes Toward Functional Foods, and Emotional Eating (Ind₁₂: X → M₁ → M₂ → M₄ → Y), was also positive and statistically significant (B = 0.03, SE = 0.01, 95% CI [0.02, 0.05]).

The thirteenth indirect effect, through e-Healthy Eating Literacy, Food Craving Acceptance and Action, and Emotional Eating (Ind₁₃: X → M₁ → M₃ → M₄ → Y), was statistically significant (B = 0.01, SE = 0.01, 95% CI [0.01, 0.01]).

The fourteenth indirect effect, through Attitudes Toward Functional Foods, Food Craving Acceptance and Action, and Emotional Eating (Ind₁₄: X → M₂ → M₃ → M₄ → Y), was positive and statistically significant (B = 0.01, SE = 0.01, 95% CI [0.01, 0.01]).

Finally, the fifteenth indirect effect, representing the full sequential mediation pathway including all mediators (Ind₁₅: X → M₁ → M₂ → M₃ → M₄ → Y), was positive and statistically significant (B = 0.01, SE = 0.01, 95% CI [0.01, 0.01]). Although the magnitude of this pathway is relatively small, its statistical significance provides evidence for the proposed multi-layered mediation mechanism, suggesting that cognitive, attitudinal, and psychological processes operate in an integrated and sequential manner in their association with eating attitudes.

As presented in Table 14, the total indirect association of Social Media and Eating Behavior with Eating Attitudes was positive and statistically significant (B = 0.51, SE = 0.04, 95% CI [0.44, 0.58]). In line with bootstrap inference, indirect effects with confidence intervals that did not include zero were considered statistically significant. Accordingly, all reported indirect pathways were significant, supporting the robustness of the mediation structure.

The direct association (c′) between Social Media and Eating Behavior and Eating Attitudes remained positive and statistically significant after controlling for all mediators, indicating partial mediation. When comparing the magnitude of the total indirect association (B = 0.51) with the direct association (c′ = 0.33), the results suggest that a substantial portion of the association is transmitted through the mediating variables.

Taken together, these findings demonstrate that the relationship between social media–related eating behavior and eating attitudes is primarily explained by indirect pathways operating through cognitive (e-Healthy Eating Literacy), attitudinal (Attitudes Toward Functional Foods), and psychological (Food Craving Acceptance and Action; Emotional Eating) mechanisms. This pattern provides strong support for the proposed serial multiple mediation model and highlights the integrated role of these processes in shaping eating attitudes.

## 4 Discussion

It is important to emphasize that the present findings are based on cross-sectional data, which precludes any definitive conclusions regarding causality or temporal ordering among variables. Although the serial mediation model is theoretically grounded, the directionality of relationships should be interpreted as hypothetical rather than causal. As highlighted by Maxwell and Cole (106), mediation analyses conducted on cross-sectional data may produce biased estimates of longitudinal processes. Therefore, the observed associations should be interpreted as consistent with the proposed theoretical framework rather than as evidence of causal pathways.

This study addresses a critical gap in the literature by examining the relationship between social media use and eating behaviors within a sequential framework encompassing cognitive (digital healthy eating literacy), attitudinal (attitudes toward functional foods), and psychological (acceptance of eating desires and emotional eating) mechanisms. The findings indicate that linear or unidimensional models do not adequately capture this relationship; rather, it reflects a multi-stage and interrelated structure. In this regard, the study supports the need for more integrative approaches to understanding the complex dynamics between social media use and eating behaviors.

The results demonstrate that social media use is significantly associated with eating behaviors, both directly and indirectly through cognitive, attitudinal, and psychological pathways. This pattern highlights the multidimensional nature of the relationship, suggesting that eating behaviors are linked to a network of interrelated processes rather than a single explanatory mechanism. These findings are consistent with prior research emphasizing the complex nature of this relationship (3, 73).

The observed association between social media use and digital healthy eating literacy represents a theoretically meaningful contribution within the framework of Social Cognitive Theory (5). Social media functions as a dynamic environment of observational learning, where norms, behavioral patterns, and health-related expectations are continuously constructed and reinforced. Within this context, individuals actively interpret and evaluate content, and outcomes depend not only on exposure but also on cognitive processing and internalization.

The findings further highlight digital healthy eating literacy as a key cognitive mechanism, enabling individuals to evaluate and utilize nutrition-related information critically. This supports the view that digital health literacy extends beyond basic knowledge, encompassing the capacity to access, evaluate, and apply information effectively (37, 39). Given the increasing reliance on social media for health information (36) and the variability in content credibility (29), this competence appears essential for informed decision-making.

The significant association between digital nutritional literacy and attitudes toward functional foods underscores the role of cognitive processes in shaping attitudinal constructs. Consistent with prior research, higher levels of literacy are associated with more favorable evaluations of food choices (43, 44). Within the Theory of Planned Behavior, attitudes are key determinants of behavioral intention (47), suggesting that individuals with higher literacy are more likely to develop positive orientations toward health-promoting foods.

The association between attitudes toward functional foods and the acceptance of eating urges highlights the intention-behavior gap. Although positive attitudes are linked to stronger intentions, they do not always translate into behavior (47). This discrepancy has been associated with impulse regulation and situational factors (48). In this context, acceptance of eating urges may function as a form of psychological flexibility, supporting more value-consistent behavior (17).

The relationship between acceptance of food-related urges and emotional eating is consistent with acceptance-based approaches such as Acceptance and Commitment Therapy. Evidence suggests that suppression-based strategies may intensify unwanted impulses, whereas acceptance-based strategies are associated with more adaptive outcomes (58, 60). The present findings indicate that acceptance processes are associated with lower levels of emotional eating, highlighting the importance of psychological regulation mechanisms.

Finally, the serial mediation analyses suggest that the relationship between social media use and eating behaviors is associated with a sequential pathway involving digital healthy eating literacy, attitudes toward functional foods, acceptance of food cravings, and emotional eating. This pattern indicates that the relationship operates through multiple interconnected processes. Given that prior studies have often relied on univariate approaches (3), the present findings offer a more comprehensive, multi-layered framework for understanding eating behavior.

## 5 Conclusion

This study, conducted among sports science students, demonstrates that the relationship between social media use and eating attitudes does not operate through a direct pathway; rather, it is associated with a multi-stage, chain-like mechanism. Specifically, the findings indicate that social media is sequentially associated with digital healthy eating literacy, attitudes toward functional foods, acceptance of food cravings, and emotional eating.

These results underscore that eating behaviors are not shaped solely by knowledge or attitudes, but are associated with the dynamic interplay of cognitive appraisal, impulse regulation, and emotional processes. Notably, digital healthy eating literacy appears to constitute a critical entry point in this mechanism, highlighting the pivotal role of how individuals interpret and process nutrition-related content encountered on social media.

In this context, interventions aimed at promoting healthy eating behaviors should go beyond mere information provision and adopt a more holistic approach. Such approaches should prioritize enhancing digital literacy, fostering favorable attitudes toward healthy and functional foods, and strengthening individuals’ capacity to manage cravings and cope with emotional eating.

## 6 Limitations

While this study’s findings make meaningful contributions to the literature, several methodological and conceptual limitations should be acknowledged.

First, the cross-sectional design limits the ability to draw causal inferences regarding the relationships among the variables. Although significant associations were identified, the directionality and temporal ordering of these relationships cannot be established. This limitation is particularly relevant for mediation analyses, as cross-sectional data restrict the interpretation of indirect pathways (106). Future research employing longitudinal or experimental designs is needed to examine the proposed sequential associations better.

Second, the sample consisted exclusively of sports science students, which may limit the generalizability of the findings. This group may differ from the general population in terms of health awareness and physical activity levels, potentially shaping the observed associations. As noted by Henrich et al. (86), findings derived from relatively homogeneous samples should be interpreted cautiously. Future studies including more diverse populations would strengthen external validity.

Third, the reliance on self-report measures may introduce bias, including social desirability and recall bias (105). Although statistical procedures indicated no serious common-method bias, these limitations cannot be fully eliminated. Future research is encouraged to incorporate objective measures, such as digital usage data or behavioral observations.

Fourth, social media use was treated as a general construct, without examining qualitative aspects such as content type, usage purpose, or platform differences. However, prior research suggests that visually rich and influencer-driven content may be more strongly associated with eating behaviors (6, 14). Future studies should incorporate these dimensions to provide a more nuanced understanding.

Fifth, the model does not include all potential determinants of eating behavior. Variables such as body image, self-control, stress, social comparison, and psychological well-being have also been linked to eating behaviors (6, 64), and their exclusion may limit the model’s explanatory scope.

Sixth, although the measurement instruments demonstrated acceptable validity and reliability, some constructs particularly emotional eating and craving acceptance are inherently complex and context-dependent (63). Future research should consider more comprehensive and multi-method assessment approaches.

Another limitation of the present study is the omission of several well-established predictors of eating behavior, such as body image dissatisfaction, social comparison tendencies, perceived stress, and self-regulation capacity. Previous research has demonstrated that these variables play a significant role in shaping eating behaviors and may interact with social media exposure (108, 109). Excluding these variables raises the possibility of omitted-variable bias, which may influence the observed relationships. Future research should incorporate these constructs to develop more comprehensive models.

Finally, the study’s cultural context is limited to Türkiye. Given that cultural norms shape both eating behaviors and social media use, the generalizability of the findings across different cultural contexts remains uncertain. Cross-cultural studies are therefore needed to evaluate the robustness of the proposed model.

Despite these limitations, the study contributes to the literature by examining the relationship between social media use and eating behaviors within a comprehensive, multidimensional framework.

## 7 Recommendations

### 7.1 Recommendations for future research

This study examines the relationship between social media use and eating behaviors within a chain mediation framework encompassing cognitive, attitudinal, and psychological processes. Based on the findings, several directions for future research can be proposed.

First, given the cross-sectional nature of the study, future research should employ longitudinal and experimental designs to examine better the temporal ordering and potential causal structure of the observed associations. While cross-sectional studies are useful for identifying relationships, they provide limited insight into underlying mechanisms (106). In particular, longitudinal designs may offer a clearer understanding of how social media use is associated with changes in eating behaviors over time.

Second, future studies should prioritize experimental and intervention-based approaches. For example, programs aimed at enhancing digital healthy eating literacy, as well as mindfulness or acceptance-based interventions, could be examined in relation to eating behaviors. Such designs would help determine whether the identified mediating mechanisms represent modifiable processes (60).

Third, as the current sample consists of sports science students, future research should include more diverse populations, such as adolescents, adults, and individuals from different socioeconomic and cultural backgrounds, to improve generalizability. Cultural differences in both social media use and eating behaviors further highlight the need for cross-cultural research (86).

Fourth, future research is encouraged to expand the analytical framework by incorporating additional variables. Constructs such as body image, social comparison, social media addiction, and hedonic hunger may provide a more comprehensive understanding of the relationship between social media use and eating behaviors (108, 110, 111).

Fifth, rather than treating social media as a homogeneous construct, future studies should examine platform-specific characteristics (e.g., Instagram, TikTok, YouTube), as visually oriented platforms may be differentially associated with eating behaviors (14).

Finally, the use of mixed-methods and qualitative designs is recommended to complement quantitative findings. Such approaches may provide deeper insights into how individuals interpret social media content and how these interpretations are associated with eating behaviors in specific contexts.

### 7.2 Practical implications

The findings of this study offer important practical implications, particularly for practitioners in nutrition, sports science, and public health.

First, the results highlight that digital healthy eating literacy is closely associated with eating behaviors. Accordingly, educational programs in universities and similar settings should aim to equip individuals with the skills to evaluate nutrition-related information in digital environments critically. Health literacy–based interventions have been associated with healthier eating patterns (38).

Second, interventions that support individuals’ ability to manage food cravings are particularly relevant. Acceptance and Commitment Therapy (ACT) and mindfulness-based approaches have been associated with lower levels of emotional eating by promoting regulation rather than suppression of cravings (58, 60). Such approaches may be especially suitable for university student populations.

Third, given that social media platforms are closely associated with health-related behaviors, their strategic use in health promotion is essential. The dissemination of evidence-based content by qualified experts may help address misinformation (30). In this context, promoting healthy eating messages, particularly through collaborations with influencers, represents a promising strategy.

Fourth, given the association between attitudes toward functional foods and eating behaviors, ensuring access to accurate, evidence-based information is critical. Educational initiatives and nutritional counseling services may support more informed food-related decisions (43).

Finally, as sports science students are potential future role models, fostering healthy eating habits within this group may contribute not only to individual well-being but also to broader societal health outcomes.

### 7.3 Implications for educational and public policy

The findings of this study offer important strategic implications for both educational practices and public policy.

First, within education policy, digital health and nutrition literacy should be systematically integrated into curricula. As individuals increasingly access health-related information through digital platforms, the ability to critically evaluate its accuracy and credibility has become an essential competency (31). Accordingly, fostering these skills across all levels of education is of key importance.

Second, from a public health policy perspective, the monitoring of nutrition-related content on social media platforms remains critical. The widespread availability of inaccurate or misleading information has been associated with unhealthy eating behaviors (30). Therefore, health authorities and regulatory bodies may adopt a more active role in guiding digital environments to improve the quality and reliability of health-related content.

Third, national campaigns promoting healthy eating may be strategically delivered through social media platforms. Given the broad reach of digital environments, these approaches are well-positioned to support population-level health communication efforts.

Fourth, public policies concerning functional foods should emphasize transparency and scientific reliability. Ensuring that product labeling, health claims, and advertising practices are grounded in robust evidence may support more informed consumer decisions (43).

Fifth, psychological and nutritional counseling services within universities should be strengthened. Emotional eating is closely associated with psychological well-being (63), highlighting the need for integrated and multidisciplinary support systems.

Finally, considering the widespread use of social media among young adults, preventive health policies targeting this population may support both individual well-being and broader public health outcomes.

## Data Availability

The original contributions presented in the study are included in the article/supplementary material, further inquiries can be directed to the corresponding authors.
